# Acidity Quantification and Structure Analysis of Amide-AlCl_3_ Liquid Coordination Complexes for C_4_ Alkylation Catalysis

**DOI:** 10.3390/molecules28237857

**Published:** 2023-11-30

**Authors:** Hao Li, Qiong Wu, Ying Liu, Jinrong Bao

**Affiliations:** School of Chemistry and Chemical Engineering, Inner Mongolia University, Hohhot 010021, China

**Keywords:** liquid coordination complexes, Lewis acidity, density functional theory, NMR, C_4_ alkylation catalysts

## Abstract

Liquid coordination complexes (LCCs), which are formed between metal halides and donor molecules, represent promising catalysts. Six amide-AlCl_3_ LCCs were successfully synthesized, followed by their characterization through NMR, Raman, and UV-visible spectroscopy. The acidity of these LCCs was quantified by performing computational modelling of fluoride ion affinities (FIA) and experimental Gutmann–Beckett measurements. Spectroscopic analysis indicated bidentate coordination between amide ligands and Al, which induced asymmetric splitting of Al_2_Cl_6_ into diverse ions such as [AlCl_2_L_2_]^+^, [AlCl_4_]^−^, [AlCl_3_L], and [Al_2_Cl_6_L]. The computed FIA was found to align well with the experimental acidity trends, thereby confirming the proposed structure of the LCC. In the alkylation tests, the LCC with a high acidity demonstrated an increase in the yields of C_5_-C_7_ alkylates. These results provide an in-depth understanding of the tuneable structures of amide-AlCl_3_ LCCs. The acidity of LCCs can be controlled by tuning the ratio of the organic ligand to AlCl_3_, which allows bidentate coordination to facilitate asymmetric splitting of Al_2_Cl_6_. The LCCs demonstrate a high degree of potential as versatile and sustainable acid catalysts in alkylation reactions. These findings may advance the foundational knowledge of LCCs for the purpose of targeted acid catalyst design.

## 1. Introduction

From photoelectron catalysis to traditional acid catalysis, liquids containing high concentrations of metals exhibit significant application value. Ideally, these liquids demonstrate exceptional control and tunable metal coordination capabilities, enabling key properties (e.g., viscosity and acidity) to be adjustable and well-regulated. Common types of high-metal-concentration liquids are molten alloys, molten salts, ionic liquids, or deep eutectic solvents [1,2]. However, these compounds exhibit several limitations, including high costs, metal solubility constraints, corrosiveness, and insufficient metal coordination capacity, hindering the realization of ideal systems. Liquid coordination complexes (LCCs) are deemed superior alternatives to ionic liquids and molten salts [3,4,5], exhibiting outstanding performance across various applications. For instance, in the alkylation of isobutane with 2-butene, LCCs prepared by combining metal halides and donor molecules possess more flexible approaches to regulating Lewis acidity and heightened catalytic activity. Additionally, these complexes are cost-effective, easily synthesized, and environmentally friendly characteristics, thus partially mitigating the drawbacks associated with ionic liquids [6,7,8].

Currently, the predominantly encountered LCCs are formed by coordinating metal halides (MCl*_x_*, *x* = 2,3) such as ZnCl_2_, SnCl_2_, CrCl_3_, FeCl_3_, AlCl_3_, and GaCl_3_ with donor molecules like urea, alcohol, and amides [1,9]. Previous investigations have demonstrated that many of these ligands induce asymmetric splitting of “M_2_Cl_6_” units [10], leading to the formation of ionized compounds like [MCl_2_L_2_][MCl_4_]. However, the intricacy of LCC complexes extends far beyond this. Recent research suggests that LCCs formed by coordinating AlCl_3_ with amides might accommodate species like [AlCl_2_(amide)*_n_*][AlCl_4_] (*n* = 1 or 2). This coordination requires the presence of [AlCl_2_(amide)_2_]^+^, [AlCl_4_]^−^, and [AlCl_2_(amide)]^+^ ions [11,12]. The presence of tridentate ions [AlCl_2_(amide)_2_]^+^ and [AlCl_2_(amide)]^+^, however, still remains a topic of contention [1,4,9,13]. Existing academic literature underscores that donor molecules possessing high dielectric constants and polarity (e.g., acetonitrile) enhance the formation of ionic materials in AlCl_3_-based LCCs, while using substances with lower dielectric constants and polarity such as tetrahydrofuran results in the formation of neutral materials [14,15]. Conversely, investigations into coordination sites and modes in amide LCCs remain quite restricted. Campos et al. found that formamide, through coordination with AlCl_3_ using the O and N atoms, can form ionic species with bidentate structures [16,17]. Hu et al. also indicated that the presence of methyl leads to bidentate coordination structures in *N*-methylacetamide-AlCl_3_ and *N*,*N*-dimethylacetamide-AlCl_3_ LCCs for both O and N atoms. In contrast, acetamide only engages monodentate O atoms to coordinate with AlCl_3_ [11,12]. The characterization of complexes and coordination sites in other amide-AlCl_3_ LCCs still presents an ambiguity. Do they form [AlCl_3_L] and [AlCl_3_L_2_], or form [AlCl_2_L_2_]^+^ and [AlCl_2_L]^+^? Do they represent monodentate or bidentate coordination structures? In light of the economical preparation and simplified synthesis routes of amide LCCs, as well as their increasing relevance in catalytic reactions, a thorough investigation of the indicated issues related to amide LCCs becomes essential.

In addition, both chloroaluminate ionic liquids and AlCl_3_-LCCs manifest significant advantages in the alkylation reaction of isobutane and 2-butene compared to traditional sulfuric acid catalysts [7,18,19,20,21]. However, accurately quantifying the acid strength of these compounds, which act as Lewis acidic catalysts, remains a challenge [22]. The International Union of Pure and Applied Chemistry (IUPAC) defines Lewis acidity as the thermodynamic propensity to form Lewis acid–base pairs, often measured by the affinity of fluoride ions [23]. The task to ascertain Lewis acidity experimentally can be complex due to the numerous bound molecules used for evaluation [24]. The Gutmann–Beckett (GB) method, a well-established method, hinges on the induced ^31^P NMR shift of triethylphosphine oxide to determine the Lewis acidity strength of a compound [25,26,27]. In general, the induced ^31^P NMR shift (*δ*_31P_) of triethylphosphine oxide can be measured to obtain the acceptor number (AN), where AN = (*δ*_31P_ − 41.0) × [100/(86.1 − 41.0)]. A higher AN value (or GB value = Δ*δ*_31P_^exp^ = *δ*_31P_^exp^ − 50) indicates a stronger Lewis acidity of the compound. On the other hand, computed fluoride ion affinity (FIA) serves as a valuable metric for evaluating Lewis acidity [28,29], although benchmark studies on the accuracy of FIA computations have been presented only to a limited extent [30]. Recently, Erdmann and Greb employed the DLPNO-CCSD(T) computational model to perform comparative FIA analysis of compounds consisting of various elements from the periodic table [23,24,29,31]. They have also studied the thermodynamic properties, nuclear magnetic resonance characteristics, and GB values of more than 130 Lewis acids. Importantly, this link between theoretical and experimental perspectives enhances our understanding of Lewis acidity in numerous compounds.

In this work, we have synthesized six amide-AlCl_3_ LCCs using a one-step approach with acetamide, propionamide, and butanamide. Additionally, chloroformamide, chloroacetamide, and 2-chloropropionamide were also used as the donor molecules. A notable contribution of this work is the establishment of a distinct correlation between the molecular structure of Lewis acidic compounds, computed fluoride ion affinity (FIA) values, and experimental Gutmann–Beckett measurements. Utilizing computed FIA and GB values, we employed hypothesis testing methods to investigate the specific molecular structures of the Lewis acidic LCCs. The six amide-LCCs were thoroughly characterized using NMR, Raman, and UV-Vis spectroscopy. These characterizations provided insights into the specific influences of amide structures on the asymmetric splitting of AlCl_3_. Altogether, this work has expanded the range of LCC catalysts and provided valuable data for the design and synthesis of the C_4_ alkylation catalysts.

## 2. Results and Discussion

### 2.1. ^27^Al NMR and Raman Spectroscopic Analysis

**Figure 1:**
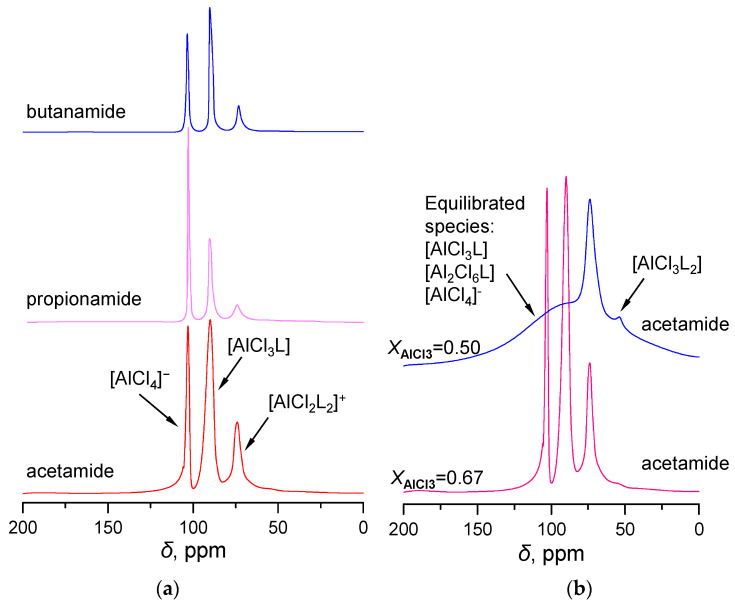
^27^Al NMR spectra of amide-AlCl_3_ systems: (**a**) butanamide-, propionamide-, and acetamide-based LCCs; (**b**) Acetamide-AlCl_3_ based on various ligands at *X*_AlCl3_ = 0.50, 0.67.

Previous studies have attributed the formation of species in amide-AlCl_3_ based LCC to the asymmetric splitting of Al_2_Cl_6_ under the induction of organic ligands (L) [1,32,33]. We sought to gather valuable insights into the structure of LCCs, recognizing that ^27^Al NMR is an effective tool for identifying aluminum species [34,35,36]. Figure 1a presents ^27^Al NMR spectra of three amide-AlCl_3_ based LCCs with a compound of ratio of *X*_AlCl3_ = 0.67.

Three distinct peaks have been identified in the ^27^Al NMR spectra of three LCCs (Figure 1a). For the LCC of acetamide-AlCl_3_, three peaks emerged at 102, 90, and 75 ppm, associated with the complexes formed between aluminum chloride and organic ligands (L, acetamide). Specifically, the downfield peak at approximately 102 ppm is attributed to the tetrahedral [AlCl_4_]^−^ anion, consistent with previous literature values. Moreover, the upfield-shifted peaks in the ^27^Al NMR spectrum are attributed to [AlCl_3_L] (90 ppm) and [AlCl_2_L_2_]^+^ (75 ppm), respectively. Notably, the [AlCl_2_L_2_]^+^ ion was distinctly detected in the NMR spectrum, absent the so-called [AlCl_2_L]^+^ ion. When *X*_AlCl3_ equals 0.5, a broad band is displayed between *δ* = 101 − 106 ppm in the acetamide-AlCl_3_ based LCC (Figure 1b). This broad signal masks the sharp signals of [AlCl_4_]^−^ and [AlCl_3_L] that were previously observed at the corresponding positions when *X*_AlCl3_ = 0.67. The observed broad band is believed to be attributed to the dynamic equilibrium of [AlCl_4_]^−^, [Al_2_Cl_6_L], and [AlCl_3_L].

**Figure 2:**
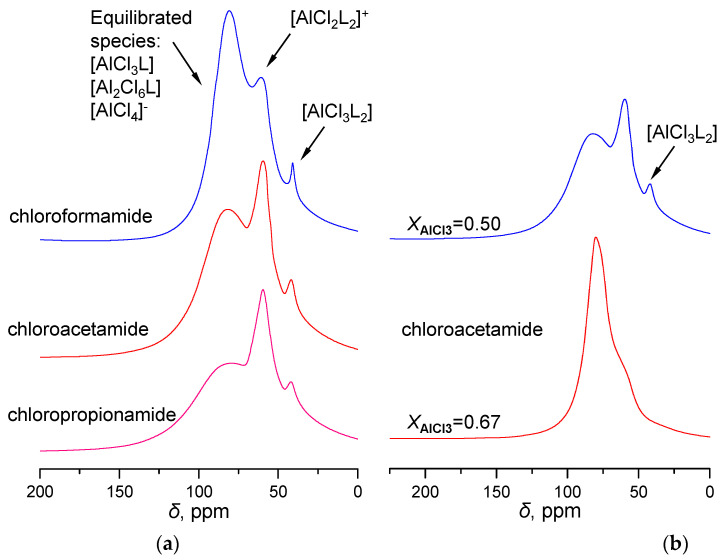
^27^Al NMR spectra of LCCs: (**a**) chloroformamide-, chloroacetamide-, and chloropropionamide-AlCl_3_ at *X*_AlCl3_ = 0.50; (**b**) chloroacetamide-AlCl_3_ based on various ligands at *X*_AlCl3_ = 0.50, 0.67.

As illustrated in Figure 2, the ^27^Al NMR spectra of the chloroamide-AlCl_3_-based LCCs were examined with *X*_AlCl3_ = 0.50. Across all the spectra of the chloroamide-based LCCs, a broad band was observed at approximately *δ* = 105 − 75 ppm. This observed band could potentially be attributed to the dynamic equilibrium between [AlCl_3_L], [Al_2_Cl_6_L], and [AlCl_4_]^−^ complexes. Moreover, the NMR spectra also identified [AlCl_2_L_2_]^+^ at the distinctive 75 ppm and [AlCl_3_L_2_] at the 52 ppm regions. These particular species exhibit intricate equilibrium relationships:

[AlCl_2_L_2_]^+^ + [AlCl_4_]^−^ + [Al_2_Cl_6_] + L ⇌ 2[AlCl_3_L] + [Al_2_Cl_6_L] (1)

[AlCl_2_L_2_]^+^ + [AlCl_4_]^−^ + 2L ⇌ 2[AlCl_3_L_2_] (2)

[AlCl_3_L_2_] + AlCl_3_ ⇌ [AlCl_2_L_2_]^+^ + [AlCl_4_]^−^ (3)

It is noted that the introduction of an organic ligand L (e.g., amide and chloroamide) or AlCl_3_ could disrupt the equilibrium of Formulas (1)−(3). This phenomenon is noticeably evident when *X*_AlCl3_ = 0.67. As the molar fraction of AlCl_3_ in acetamide-LCC increases from 0.50 to 0.67 (Figure 2b), a substantial alteration is manifested in the [AlCl_2_L_2_]^+^ signal peak of the acetamide-AlCl_3_-based LCC, whereas the [AlCl_3_L_2_] peak virtually vanishes. This suggests that an increase in AlCl_3_ in LCC fosters the rightward shift of the reaction equation (3). The NMR spectra of chloroacetamide-AlCl_3_ provides further evidence supporting this inference. Upon increasing the *X*_AlCl3_ proportion from 0.50 to 0.67, the [AlCl_3_L_2_] signal peak becomes unobservable. Simultaneously, the signal peak resulting from the equilibrated species [AlCl_4_]^−^, [Al_2_Cl_6_L], and [AlCl_3_L] exhibits a progressive broadening.

**Figure 3:**
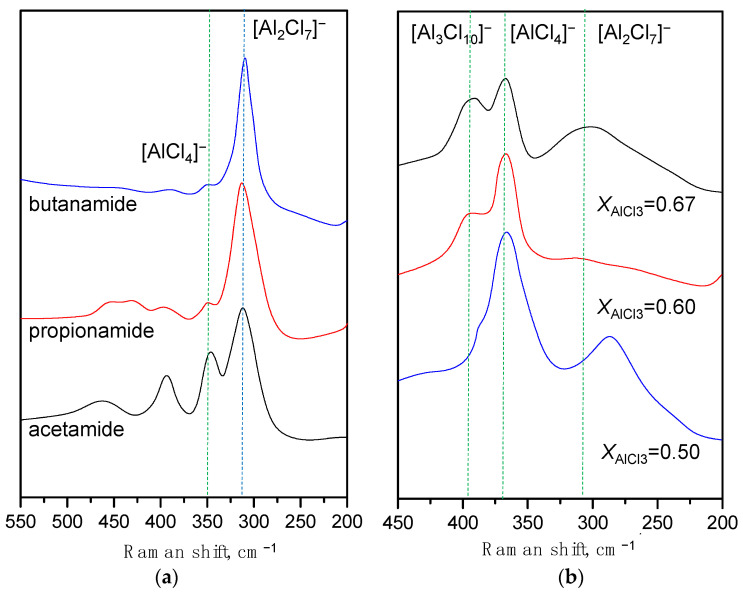
Raman spectra: (**a**) amide-AlCl_3_-based LCCs with *X*_AlCl3_ = 0.67; (**b**) chloroacetamide-AlCl_3_-based LCC with dominant bands for Al-Cl stretching frequencies assigned to chloroaluminate anions.

The presence of broad bands in the ^27^Al NMR spectrum obscures the specific structure of Al species in LCCs. In particular, previous studies have consistently suggested that the signal of [Al_2_Cl_7_]^−^ cannot be identified through ^27^Al NMR spectroscopy [3,36,37]. Nevertheless, this limitation can be mitigated by employing Raman spectroscopy. The Raman spectra of amide-based LCCs exhibit distinct peaks corresponding to [AlCl_4_]^−^ (350 cm^−1^) and [Al_2_Cl_7_]^−^ (315 cm^−1^) within the range of 400–300 cm^−1^ (Figure 3a) [5,38,39]. There is a decrease in the intensity of [AlCl_4_]^−^ associated with an increase in alkyl group size. That is, the reaction of 2[AlCl_4_]^−^ ⇌ [Al_2_Cl_7_]^−^ + Cl^−^ is more likely to be influenced towards the right by smaller organic ligands. On the Raman spectrum of chloroacetamide-AlCl_3_ (Figure 3b), a shoulder related to [Al_3_Cl_10_] appears when *X*_AlCl3_ = 0.60 (390 cm^−1^). Upon increasing the *X*_AlCl3_ to 0.60 and 0.67, this shoulder develops into a prominent band.

### 2.2. Experimental Measurement of LCC Acidity

The purpose of the study on LCC was to catalyze the C_4_ alkylation reaction between isobutane and 2-butene. However, this reaction requires a high acid strength. According to the Hammett acidity function, only H_0_ values lower than −10 can initiate the reaction [40]. Conversely, excessive acidity can result in increased side reactions in alkylation, thus lowering the quality of alkylate gasoline. The industrial evaluation of acid strength in alkylation catalysts primarily focuses on Brönsted acidity, with relatively few studies dedicated to the acid strength of Lewis acidic catalysts [18]. The Gutmann method can be used to measure Lewis acid strength by quantifying the acceptor number (AN) of a compound through the measurement of the ^31^P NMR chemical shift of a basic probe molecule, triethylphosphine oxide (TEPO) [26]. However, the Gutmann method is unwieldy, because it necessitates the extrapolation of data for TEPO-Lewis acid [41]. To simplify the measurement, the Gutmann–Beckett method has been developed [27]. This method enables the quantification of Lewis acidity by measuring the GB value (Δ*δ*_31P_^exp^). The GB value is positively correlated with Lewis acid strength, and it can serve to quantify the acid strength of LCCs and to some extent infer the molecular structure of the Lewis acid.

The ^31^P NMR spectroscopic analysis revealed that the AN values of LCCs were much higher than those of chloroaluminate ionic liquids with *X*_AlCl3_ = 0.50 (e.g., [BMIm]Cl-AlCl_3_ and [(C_2_H_5_)_3_NH]Cl-AlCl_3_). Generally, when *X*_AlCl3_ = 0.50, the chloroaluminate ionic liquid does not exhibit acidity. Notably, the product of acetamide-AlCl_3_ contains a significant proportion of C_8_ olefins, reaching up to 84.1%. This high yield is a result of acid-catalyzed oligomerization of C_4_ olefins, suggesting that LCCs exhibit Lewis acidity due to the asymmetric splitting of Al_2_Cl_6_ when *X*_AlCl3_ = 0.50. In addition, the Gutmann–Beckett method was used to determine the AN values (AN = (*δ*_31P_ − 41.0) × [100/(86.1 − 41.0)]) of the six studied LCCs with *X*_AlCl3_ = 0.67, as shown in Table 1. The AN values of the LCCs were higher than those of [(C_2_H_5_)_3_NH]Cl-AlCl_3_ and [BMIm]Cl-AlCl_3_. Particularly, the AN values of three chloroamide-AlCl_3_-based LCCs were approximately 100 (98–102), indicating that the LCCs have significant potential for use in Lewis acid catalysis. Moreover, the results of the alkylation of the six LCC catalysts are shown in Table 1.

### 2.3. DFT Calculations of FIA Values 

NMR and Raman spectroscopy measurements, as well as the determination of AN values, have provided a general direction for the structural determination of LCCs. However, considering the difficulties with experimental determination of the structures of liquid catalysts, it is necessary to obtain additional information from theoretical calculations. The calculation of fluoride ion affinity (FIA) is a valuable descriptor for evaluating the strength of Lewis acids in compounds. Despite its widespread use, a scarcity of data and comparative studies for larger Lewis acids impedes the broad comparability between experimental results and calculated data.

LA + COF_3_^−^ → [LA-F]^−^ + COF_2_    ΔH_1_, LA is Lewis Acid. (4)

COF_3_^−^ → COF_2_ + F^−^      ΔH_2_ (experimental 208.8 kJ·mol^−1^) (5)

LA + F^−^ → [LA-F]^−^      FIA = ΔH_1_ − ΔH_2_ (6)

Herein, we have evaluated the performance of selected methods (e.g., DLPNO-CCSD(T)) for FIA computation based on CCSD(T)/CBS data and Erhmann’s benchmark procedure [31]. The FIA values for the studied LCCs and chloroaluminate ionic liquids have been calculated. The computed FIA values can be compared unbiasedly against each other (Table S2), and these values provide a critical benchmark for evaluating the acid strength of prospective alkylation catalysts. Table 2 presents the computed FIA and GB values for the six studied LCCs.

The results indicate a definitive correlation between the FIA values and GB values among the six LCCs. Specifically, as the computed FIA value increases, the GB value also tends to increase. Butanamide-AlCl_3_ and chloropropionamide^-^AlCl_3_ exhibit relatively high FIA/GB values. The larger GB values imply a more pronounced ability to accept fluoride ions, indicating stronger Lewis acidity. This is consistent with the earlier evaluation results of alkylation experiments, where chloropropionamide^-^AlCl_3_ showed higher catalytic activity. Within Table 1, the C_5_–C_7_ fractions of these catalysts are significantly greater than those of other LCCs. Additionally, the GB values of amide- or chloroamide-LCCs decrease as the molecular weight of the derivatives increases, suggesting a decrease in overall acidity due to coordination between Lewis acidic species and larger organic ligands.

However, we have noted that the FIA values vary when calculations are conducted on varied structures of the LCC. As depicted in Table 2, calculations of the same LCC employing different species structures yield varying FIA values. The diverse FIA values associated with a compound lead to multiple possibilities for material structure analysis, yet they also introduce some degree of confusion. Therefore, it is necessary to further clarify the relationship between FIA, GB, and species structures.

### 2.4. Computed FIA, Δδ_31P_^exp^, and Structure

Based on the dataset provided by Erdmann and Greb [24], a strong correlation was found between the calculated FIA, Δ*δ*_31P_^exp^, and the molecular structure of Lewis acids containing aluminum (Tables S1 and S2). Accurate FIA values rely on precise molecular structures of Lewis acids. According to the existing GB-FIA relationship, the experimental Δ*δ*_31P_^exp^ (GB value) of a new Lewis acid can be used to estimate the FIA value. By comparing the residuals between the predicted FIA and the calculated FIA values with the confidence interval of the GB-FIA model, the accuracy of the molecular structure associated with the calculated FIA can be determined. Step 1: establish a GB-FIA regression model based on previous FIA and experimentalΔ*δ*_31P_^exp^ values. Step 2: calculate the residuals between FIA and the predicted FIA usingΔ*δ*_31P_^exp^. Step 3: measure the *Δδ*_31P_^exp^ of the new Lewis acid molecule in advance and calculate the FIA value based on its optimized structure. Step 4: perform a hypothesis test to verify the correctness of FIA and the molecular structure for the new Lewis acid molecule. H_0_: the new FIA value conforms to the GB-FIA model. H_1_: the calculated FIA value of the new Lewis acid does not conform to the GB-FIA model, indicating that the molecular structure constructed during the FIA calculation does not match the actual situation. For more details, please see the Supplementary Materials.

**Figure 4:**
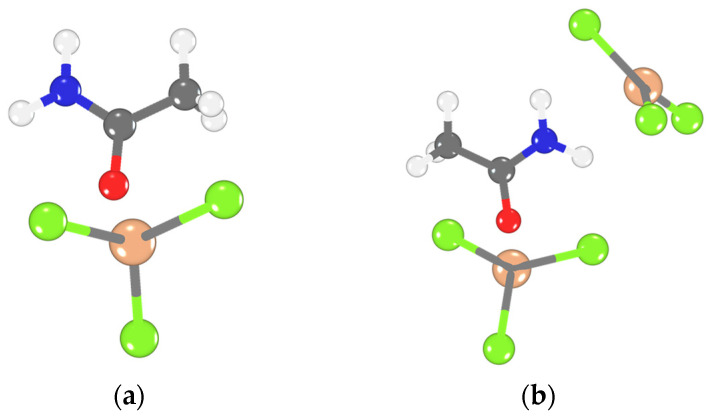
(**a**) Monodentate coordination; (**b**) bidentate coordination of acetamide−AlCl_3_. Element color: C gray, Cl green, Al orange, O red, N blue, and H light gray.

Based on the aforementioned hypothesis tests, we focused on analyzing the relationship between the structures, FIA, and GB values of six amide-based LCCs (Table 3). Specifically, in the case of acetamide-LCC at *X*_AlCl3_ = 0.5, AlCl_3_ coordinates with acetamide through a monodentate site via the O atom. Among the various configurations of the AlCl_3_-acetamide coordination relationship, only the monodentate coordination model exhibits a significant correlation between the computed FIA and the predicted FIA from the experimental GB value (426 vs. 427 in Table 3). As *X*_AlCl3_ increases to 0.67, it is more likely that AlCl_3_ coordinates with the organic ligand through both the O and N atoms of acetamide in a bidentate coordination manner (Figure 4). Clear evidence supporting the aforementioned results is illustrated in the model structures that demonstrate a consistent match between the computed FIA and GB values.

**Figure 5:**
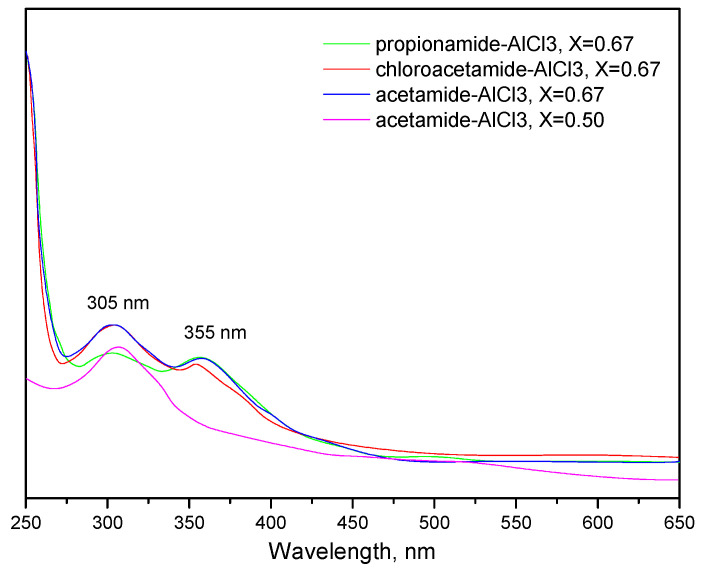
UV—vis spectra of LCCs (1 wt.%) in the dichloromethane solution.

Further investigation shows that regardless of whether *X*_AlCl3_ is 0.5 or 0.67, most amide- and chloroamide-based ligands are likely to coordinate with Al species through bidentate coordination. This appears to be the only manner to obtain a match between the calculated FIA values and the experimental GB values. More definitive evidence is provided by the UV-visible spectra of LCCs (Figure 5). In the UV-Vis spectrum of acetamide-AlCl_3_ (*X*_AlCl3_ = 0.50), a peak at 305 nm can be identified. This peak is attributed to ligand-metal charge transfer (LMCT) absorption, induced by coordination between the O atoms and Al atoms. No further significant absorption peaks are observed, indicating that acetamide-AlCl_3_ (*X*_AlCl3_ = 0.50) primarily exists in monodentate coordination. In the UV-Visible spectra of other LCCs, two LMCT absorption peaks are identified at 305 and 355 nm, indicating coordination between N and O atoms with Al atoms. This corresponds to the primary absorption peaks in a bidentate coordination structure. The steric hindrance from the larger alkyl groups or the Cl in the chloroamide leads to an excess of Al species coordinating not only with the O atom, but also the N atom. This provides a possible explanation for the occurrence of monodentate coordination when the *X*_AlCl3_ proportion in acetamide-AlCl_3_ is 0.50. On the other hand, when there is a high concentration of Al species with *X*_AlCl3_ equal to 0.67 within acetamide-AlCl_3_, the predominant form is bidentate coordination. Moreover, these results suggest that bidentate coordination is more favorable for the asymmetric splitting of Al_2_Cl_6_ than monodentate coordination. This might be the primary reason for the various forms of Al species in LCCs.

## 3. Materials and Methods

### 3.1. Materials

All reagents were used as received without any further purification, unless otherwise stated. Sodium aluminate (99%) and dichloromethane (99%) were purchased from Sigma-Aldrich Company. They were dried over 3 Å molecular sieves prior to use and stored in a glove box. Acetamide (99.5%), propionamide (99.5%), butanamide (99.5%), chloroformamide (99%), chloroacetamide (99%), 2-chloropropionamide (99%), and anhydrous aluminum chloride (99.5%) were obtained from Aladdin Chemistry Company (Shanghai, China).

### 3.2. Preparation of LCCs

Under N_2_ protection, LCCs with different molar ratios of amides to aluminum chloride were prepared in a glove box. The synthesis process of acetamide-AlCl_3_ (*X*_AlCl3_ = 0.67) is outlined as follows. A 150 mL three-neck flask equipped with a constant temperature oil bath and a stirrer was used for the synthesis. Anhydrous AlCl_3_ (13.34 g, 0.10 mol) was first added to the flask, followed by the slow addition of acetyl amide (2.96 g, 0.05 mol) while stirring for 20 min. The mixture was then heated to 90 °C and stirred for 4 h until all solids were completely dissolved, resulting in a liquid phase. The obtained LCCs were stored in the glove box for further use.

### 3.3. Characterization

The ^27^Al NMR spectrum was obtained using the Bruker Avance spectrometer, with a resonance frequency of 160.15 MHz at 25 °C. During the measurement, a 1.1 M solution of Al(NO_3_)_3_ dissolved in D_2_O was used as the external reference for the ^27^Al NMR chemical shift. Neat 85% H_3_PO_4_ was used as the reference solution for ^31^P NMR (to determine the GB value, or AN value). The LCC sample was placed in a standard 10 mm tube, with capillaries containing H_3_PO_4_ and Al(NO_3_)_3_/D_2_O inserted at the center. The measurement of AN value was performed as described in Ref. [41]. Raman spectroscopy measurements were carried out using the Perkin-Elmer Frontier 200 spectrometer. To prevent water contamination, the LCC sample was placed in a tightly sealed vial. The excitation light from the argon ion laser at 532 nm had an emission power of approximately 300–750 mW. The optical resolution of the Raman spectrum was 1 cm^−1^, with a wave number reproducibility of 0.2 cm^−1^. The ultraviolet-visible spectrum was recorded using a Shimadzu UV-2550 spectrophotometer in a quartz cell with a length of 0.1 cm. The UV-Visible spectrum was measured using a Shimadzu UV-2600 spectrophotometer in a quartz cell with a length of 0.1 cm pathway.

### 3.4. Computed FIA

The computed FIA values followed the procedure described by Ref. [31]: (1) The geometry optimization of all structures was performed using the PBEh-3c/def2-mSVP method implemented in the ORCA 5.04 software package [42]. It was confirmed that the optimized structures corresponded to energy minima on the potential energy surface by conducting analytical calculations of the PBEh-3c harmonic frequencies. (2) Single-point energy calculations were carried out using the DLPNO-CCSD(T)/aug-cc-pVQZ method [43] to obtain the electronic energy of both Lewis acid (LA) and [LA+F]^−^ structures. The total enthalpy of the Lewis acids and their fluorinated adducts was determined by combining the electronic energy obtained in this step with the thermal corrections from the previous geometry optimization. (3) To obtain the absolute value of FIA, the molecular structure of the selected reference system (COF_2_/COF_3_^−^) was also optimized using the same method as in the first step. Subsequently, single point energy calculations were performed using the same method as in the second step. (4) The reaction enthalpy for the equation LA + COF_3_^−^ → [LA-F]^−^ + COF_2_ was calculated. The experimental FIA value [44] of COF_2_ (208.8 kJ·mol^−1^) was subtracted from the calculated value. The final computed FIA values can show an accuracy within kJ·mol^−1^.

### 3.5. The C_4_ Alkylation Reaction

**Figure 6:**
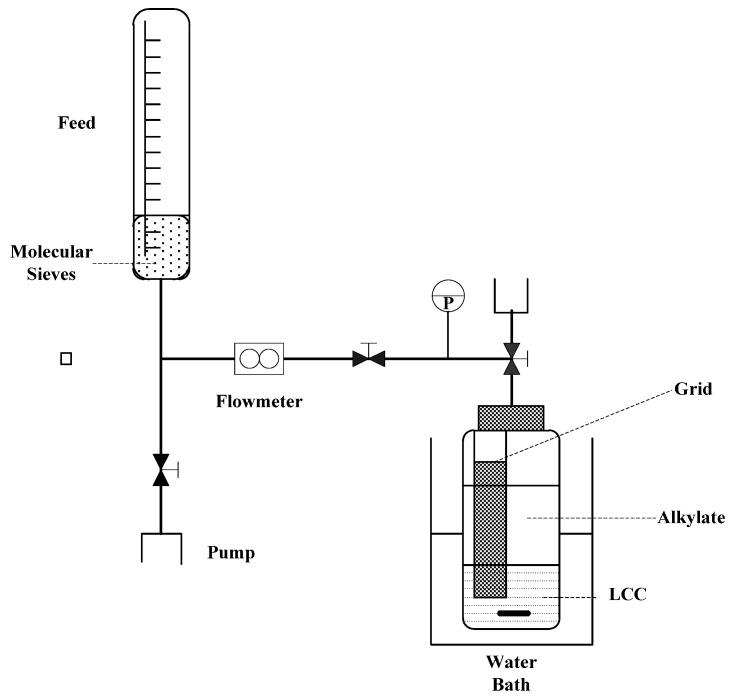
Scheme of the C_4_ alkylation reaction.

The isobutane/2-butene (C_4_) alkylation reaction was conducted in a 250 mL pressure-resistant reactor with a water bath temperature of (25 ± 0.5 °C) [45]. The stirring speed was maintained at 1000 rpm. Initially, the LCC was loaded into the reactor, followed by the addition of the C_4_ mixture. The molar ratio of isobutane to olefin in the feed was 5:1. Figure 6 illustrates the schematic diagram of the alkylation reaction. The reaction time for alkylation was 30 min. After completion, the reaction product and catalyst were poured out of the reactor and allowed to settle for approximately 20 min. To remove excess isobutane, the product was subjected to fractional distillation in a distillation column equipped with a reflux section. The product distribution was analyzed using gas chromatography (HP6890, PONA, 50 m capillary column).

## 4. Conclusions

This work experimentally measures the Lewis acidity of amide-AlCl_3_ liquid coordination complexes (LCCs), and also demonstrates the method of calculating the acidity of LCCs by constructing molecular models. The LCCs exhibit their potential as versatile and environmentally friendly catalysts for C_4_ alkylation reactions. The acidity of LCC was precisely quantified by determining the fluoride ion affinity and referencing the Gutmann–Beckett acidity scale observed in ^31^P NMR spectroscopic analysis, which also facilitated the definitive confirmation of the molecular structures of LCCs. The calculations revealed that increasing the AlCl_3_ ratio promotes formation of [AlCl_2_L_2_]^+^ and [AlCl_4_]^−^ ions while decreasing [AlCl_3_L_2_], correspondingly increasing LCC acidity. When evaluated in the alkylation of isobutane with 2-butene, the LCCs with the highest acid strength produced a higher yield of C_5_-C_7_ alkylate hydrocarbons, confirming their potential as adjustable acid catalysts for the reactions. The correlation developed between computational and experimental acidity measurements provides a valuable guide for designing new LCCs with targeted acidity for catalytic applications. In summary, the combination of experimental and computational methods for measuring acidity allows for precise adjustment of LCCs to achieve optimal acid strength and catalytic activity for environmentally friendly alkylation processes.

## Figures and Tables

**Table 1:** AN values and C4 alkylation performances of Lewis acid systems.

No.	Catalysts	AN	*X*_AlCl3_	Composition of Alkylate, wt%		
				C_5–7_	C_8_ ^1^	C_9+_
1	[(C_2_H_5_)_3_NH]Cl−AlCl_3_	70.7	0.50	-	-	-
2	[(C_2_H_5_)_3_NH]Cl−AlCl_3_	92.2	0.67	30.2	34.2	35.6
3	[BMIm]Cl−AlCl_3_	91.7	0.67	23.5	42.0	34.5
4	acetamide−AlCl_3_	82.2	0.50	2.2	84.1 ^2^	13.7
5	butanamide−AlCl_3_	96.4	0.67	20.4	45.6	34.0
6	propionamide−AlCl_3_	97.0	0.67	20.2	48.3	31.5
7	acetamide−AlCl_3_	97.6	0.67	20.1	43.4	36.5
8	chloropropionamide−AlCl_3_	98.2	0.67	25.4	48.2	26.4
9	chloroacetamide−AlCl_3_	99.1	0.67	24.2	49.6	26.2
10	chloroformamide−AlCl_3_	102.0	0.67	37.5	44.7	17.8

^1^ C_8_ include trimethyl-pentane and dimethyl-hexane; ^2^ Herein, C_8_ are mainly the mixture of C_8_ olefins.

**Table 2:** GB and computed FIA values.

Lewis Acids	GB	Constructed Species Model, FIA		
		I	II	III
[(C_2_H_5_)_3_NH]Cl−AlCl_3_ ^1^	22.9	(C_2_H_5_)_3_NH^+^/AlCl_4_^−^, 345		
[(C_2_H_5_)_3_NH]Cl−AlCl_3_	32.6	(C_2_H_5_)_3_NH^+^/Al_2_Cl_7_^−^, 500		
[BMIm]Cl−AlCl_3_	32.4	BMIm^+^/Al_2_Cl_7_^−^, 490		
acetamide−AlCl_3_ ^1^	23.1	AlCl_2_L_2_^+^/AlCl_4_^−^, 426	AlCl_3_L_2_/AlCl_3_L/Al_2_Cl_6_L, 517	
butanamide−AlCl_3_	34.5	AlCl_2_L_2_^+^/Al_2_Cl_7_^−^, 505	AlCl_3_L/Al_2_Cl_6_L, 531	AlCl_3_L_2_/AlCl_3_L/Al_2_Cl_6_L, 589
propionamide−AlCl_3_	34.8	AlCl_2_L_2_^+^/Al_2_Cl_7_^−^, 515	AlCl_3_L/Al_2_Cl_6_L, 539	AlCl_3_L_2_/AlCl_3_L/Al_2_Cl_6_L, 594
acetamide−AlCl_3_	35.0	AlCl_2_L_2_^+^/Al_2_Cl_7_^−^, 520	AlCl_3_L/Al_2_Cl_6_L, 550	AlCl_3_L_2_/AlCl_3_L/Al_2_Cl_6_L, 603
chloropropionamide−AlCl_3_	35.3	AlCl_2_L_2_^+^/Al_2_Cl_7_^−^, 530	AlCl_3_L/Al_2_Cl_6_L, 538	AlCl_3_L_2_/AlCl_3_L/Al_2_Cl_6_L, 600
chloroacetamide−AlCl_3_	35.7	AlCl_2_L_2_^+^/Al_2_Cl_7_^−^, 547	AlCl_3_L/Al_2_Cl_6_L, 580	AlCl_3_L_2_/AlCl_3_L/Al_2_Cl_6_L, 602
chloroformamide−AlCl_3_	37.0	AlCl_2_L_2_^+^/Al_2_Cl_7_^−^, 575	AlCl_3_L/Al_2_Cl_6_L, 603	AlCl_3_L_2_/AlCl_3_L/Al_2_Cl_6_L, 616

^1^ *X*_AlCl3_ = 0.50, and *X*_AlCl3_ = 0.67 for the other Lewis acids.

**Table 3:** Relationships of GB, computed FIA, and predicted FIA values.

Acids	FIA ^1^	GB	LCC	GB	Predicted FIA ^2^	Computed FIA ^3^
AlMe_3_	368	16.7	acetamide−AlCl_3_ ^4^	23.1	427	426
AlEt_3_	379	15.9	butanamide−AlCl_3_	34.5	593	589
[AlCalixMe]^−^	402	18.5	propionamide−AlCl_3_	34.8	595	594
AlN(OC_7_H_4_)_3_	412	21.2	acetamide−AlCl_3_	35.0	597	603
AlCl_3_	565	32.2	chloropropionamide−AlCl_3_	35.3	600	599
AlBr_3_	577	33.1	chloroacetamide−AlCl_3_	35.7	604	602
AlI_3_	582	34.4	chloroformamide−AlCl_3_	37.0	616	615
Al(C_6_F_5_)_3_	596	36				

^1^ Ref. [24]; ^2^ FIA values were predicted by the regression model. ^3^ FIA values were calculated by the DFT method. ^4^ *X*_AlCl3_ = 0.50, and *X*_AlCl3_ = 0.67 for the other LCCs.

## Data Availability

Data are contained within the article and Supplementary Materials.

## Supplementary Materials

The following supporting information can be downloaded at: https://www.mdpi.com/article/10.3390/molecules28237857/s1, Figure S1: The correlation model established by Al-compound GB-FIA database; Table S1: Collection of literature known and measured Gutmann–Beckett values (Δ*δ*_31P_^exp^); Table S2: Computed FIA values of Lewis acids given in kJ/mol^−1^; Program code: Program for establishing the GB-FIA model and hypothesis test.
