# Supplementary material for: Acidity Quantification and Structure Analysis of Amide-AlCl3 Liquid Coordination Complexes for C4 Alkylation Catalysis
Source: Molecules. 2023 Nov 30;28(23):7857. doi: 10.3390/molecules28237857 (PMC10708379; doi:10.3390/molecules28237857)

Supplementary Materials

Acidity Quantification and Structure Analysis of Amide-$AlCl_3$ Liquid Coordination Complexes for C_4 Alkylation Catalysis

Hao Li †, Qiong Wu †, Ying Liu * and Jinrong Bao *

School of Chemistry and Chemical Engineering, Inner Mongolia University, Hohhot 010021, China
* Correspondence: celiuy@imu.edu.cn (Y.L.); jinrongbao@imu.edu.cn (J.B.).
† These authors contributed equally to this work.

1. Gutmann-Beckett values and computed FIA values.

Table S1. Collection of literature known and measured Gutmann-Beckett values ($\Delta\delta_{31P}^{exp}$) [23].

Lewis Acid	$\Delta\delta_{31P}^{exp}$ [ppm]
BH_3	33.1
BF_3	29.0
BCl_3	38.7
BBr_3	40.3
BI_3	42.9
BEt_3	1.9
BPh_3	20.6
$B(C_6F_5)_3$	27.0
$B(C_6F_5)_2(C_6Cl_5)$	25.8
$B(C_6F_4\text{-}o\text{-}C_6F_5)_3$	30.7
$B(NMe_2)_3$	-4.9
$B(O\mathit{i}Pr)_3$	0.8
$B(OMe)_3$	-1.9
$B(OC_6F_5)_3$	30.9
$B(OC_6H_5)_3$	19.4
Cl-B(cat)	30.5
Cl-B(nad)	31.5
Ph-B(cat)	19.2
Ph-B(nad)	17.9
C_6F_5-B(cat)	26.6
C_6F_5-B(nad)	27.5
Me_3-B(cat)	-3.5
Me_3-B(nad)	-3.7
F_2-B(cat)	23.9
F_2-B(nad)	23.6
F_3-B(cat)	24.9
F_3-B(nad)	23.8
Cl_2-B(cat)	20.2
Cl_2-B(nad)	15.2
9-B(Trypt)	25.0
$AlCl_3$	32.2
$AlBr_3$	33.1 / 35.4
AlI_3	30.8 / 37.4
$AlMe_3$	16.7
$AlEt_3$	15.9
$Al(C_6F_5)_3$	26.0

$AlN(OC_7H_4)_3Cl$	21.2
$AlN(OC_7H_4)_3Me$	19.1
[AlCalixMe]-	18.5
$Ga(C_6F_5)_3$	23.1
$GaN(OC_7H_4)_3Cl$	22.1
$GaN(OC_7H_4)_3Me$	19.6
$GaCl_3$	31.1
GaI_3	28.3 / 30.2
$InCl_3$	1.5 / 13.7
$InBr_3$	3.2 / 20.3
$In(C_6F_5)_3$	19.4
$SiCl_4$	25.1 / 25.0
$SiBr_4$	37.3 / 37.4
SiI_4	58.1 / 58.2
1-Si(catH)$_2$	33.2
2-Si(catH)$_2$	17.7
1-Si(catF)$_2$	36.6
2-Si(catF)$_2$	22.2
1-Si(catCl)$_2$	37.2
2-Si(catCl)$_2$	23.1
2-Si(catBr)$_2$	37.3
2-Si(catBr)$_2$	23.1
1-Si(cattBu)$_2$	31.6
2-Si(cattBu)$_2$	20.5
1-Si(catCF$_3$)$_2$	39.4
2-Si(catCF$_3$)$_2$	24.7
$[SiEt_3]^+$	38.6
$[Si(C_6Me_6)_3]^+$	35.4
[Si(Xylyl)$_3$]$^+$	38.5
[Si(Tipp)$_3$]$^+$	41.1
[Si(Tipp)$_2$Et]$^+$	41.3
$[Si(NMe_2)_3]^+$	35.2
2-GeF_4	26.4
2-$GeCl_4$	10.1
1-Ge(catCl)$_2$	33.7
2-Ge(catCl)$_2$	20.6
[Ge(Tipp)$_2$Me]$^+$	36.6
[NBn-BZIMPYGeCl]$^+$	41.9
$SnCl_4$	17.4
SnI_4	17.9 / 17.7
[Sn(iPr)$_3$]$^+$	21.7
[Sn(nBu)$_3$]$^+$	20.0
SnCalixEt	19.3
[NBn-BZIMPYSn]$^{2+}$	26.5
[Ph-CAAN]$^+$	6.5
$[P(C_6F_5)_3F]^+$	41.1
[O=P-CDipp]$^+$	6.3
[O=P-NDipp]$^+$	37.2
[Ph-P(bipy)]$^{2+}$	34.0
[Ph-P(terpy)]$^{2+}$	5.0
[(terpy)PPh]$^{2+}$	5.8
[P(η_5-Cp*)]$^{2+}$	64.7
$AsCl_3$	3.4 / 7.6

AsI_3	13.1 / 22.0
$[As(\eta_5\text{-}Cp^*)]_2^+$	49.6
SbF_3	7.2 / 13.0
$SbCl_5$	36.1
$SbCl_3$	18.1 / 21.2
$Sb(Mesityl)_2OTf$	31.6
$Sb(Mesityl)_2Cl$	1.0
Sb-NNN	-2.2
$[Sb(\eta_5\text{-}Cp^*)]_2^+$	39.0
$BiCl_3$	16.0 / 25.9
$BiBr_3$	11.5 / 24.3
$Bi(Mesityl)_2OTf$	14.6
$Bi(Mesityl)_2Cl$	5.3
Bi-NNN	3.6
$[S(OFPh_2)]^+$	34.5
$[F\text{-}I(Ph)]^+$	40.3
$[Cl\text{-}I(Ph)]^+$	35.6
$[C_6F_5\text{-}I(Ph)]^+$	13.1
ZnF_2	0.6 / 0.5
$ZnCl_2$	18.6 / 20.4
$ZnBr_2$	18.5 / 20.2
ZnI_2	18.3 / 18.3
$Zn(C_6F_5)_2$	17.7
$[ZnCDipp(C_6F_5)]^+$	25.2
$[ZnCDippMe]^+$	10.0
$TiCl_4$	22.7

Table S2. Computed FIA values of Lewis acids given in kJ/mol^{-1} [23] (computed on DLPNO-CCSD(T)/aug-cc-pVQZ level of theory according to Ref. [24]).

**Lewis Acid**	**FIA**
BEt_3	264
$B(OMe)_3$	215
$B(OiPr)_3$	220
$B(NMe_2)_3$	168
$B(OC_6H_5)_3$	323
$B(C_6F_5)_2(C_6Cl_5)$	422
$B(C_6F_4\text{-}o\text{-}C_6F_5)_3$	449
Cl-B(cat)	352
Cl-B(nad)	373
Ph-B(cat)	424
Ph-B(nad)	355
C_6F_5-B(cat)	378
C_6F_5-B(nad)	387
Me_3-B(cat)	310
Me_3-B(nad)	308
F_2-B(cat)	357
F_2-B(nad)	423
F_3-B(cat)	417
F_3-B(nad)	443
Cl_2-B(cat)	368
Cl_2-B(nad)	348
$_9$-B(Trypt)	444

$AlEt_3$	379
$AlN(OC_7H_4)_3$	412
$AlN(OC_7H_4)_3$	363
[AlCalixMe]-	132
$GaN(OC_7H_4)_3$	351
$GaN(OC_7H_4)_3$	291
$InCl_3$	426
$InBr_3$	419
$In(C_6F_5)_3$	420
2-$SiCl_4$	-28
2-$SiBr_4$	17
2-SiI_4	60
1-$Si(catH)_2$	395
2-$Si(catH)_2$	-93
1-$Si(catF)_2$	483
2-$Si(catF)_2$	9
1-$Si(catCl)_2$	490
2-$Si(catCl)_2$	42
1-$Si(catBr)_2$	469
2-$Si(catBr)_2$	52
1-$Si(cattBu)_2$	367
2-$Si(cattBu)_2$	-172
1-$Si(catCF_3)_2$	570
2-$Si(catCF_3)_2$	127
1-SiCalixEt	484
2-SiCalixEt	56
$[SiEt_3]^+$	934
$[Si(C_6Me_6)_3]^+$	782
$[Si(Xylyl)_3]^+$	838
$[Si(Tipp)_3]^+$	732
$[Si(Tipp)_2Et]^+$	746
$[Si(NMe_2)_3]^+$	829
1-$Si(cattBu)_2$	367
2-$Si(cattBu)_2$	-172
1-$Si(catCF_3)_2$	570
2-$Si(catCF_3)_2$	127
1-SiCalixEt	484
2-SiCalixEt	56
$[SiEt_3]^+$	934
$[Si(C_6Me_6)_3]^+$	782
$[Si(Xylyl)_3]^+$	838
$[Si(Tipp)_3]^+$	732
$[Si(Tipp)_2Et]^+$	746
$[Si(NMe_2)_3]^+$	829
2-GeF_4	-142
2-$GeCl_4$	-47
1-$Ge(catCl)_2$	490
2-$Ge(catCl)_2$	60
$[Ge(Tipp)_2Me]^+$	753
$[NBn\text{-}BZIMPYGeCl]^+$ 549	120
2-$SnCl_4$	-17
2-SnI_4	17
$[Sn(iPr)_3]^+$	786

$[Sn(nBu)_3]^+$	893
1-SnCalixEt	430
2-SnCalixEt	79
$[NBn\text{-}BZIMPYSn]^{2+}$	938
PI_3	298
$[P(C_6F_5)_3F]^+$	723
$[O{=}P\text{-}CDipp]^+$	628
$[O{=}P\text{-}NDipp]^+$	666
P-NNN	302
$[Ph\text{-}P(bipy)]^{2+}$	815
$[Ph\text{-}P(terpy)]^{2+}$	872
$[(terpy)PPh]^{2+}$	796
$[P(\eta^5\text{-}Cp^*)]^{2+}$	1167
AsI_3	289
As-NNN	313
$[As(\eta^5\text{-}Cp^*)]^{2+}$	1123
$Sb(Mesityl)_2Cl$	241
$Sb(Mesityl)_2OTf$	309
Sb-NNN	386
$BiCl_3$	320
$BiBr_3$	338
$Bi(Mesityl)_2Cl$	257
$Bi(Mesityl)_2OTf$	282
Bi-NNN	311
$[S(FOPh_2)]^+$	616
$[S(FObiph)]^+$	611
[F-I(Ph)]	776
$[Cl\text{-}I(Ph)]^+$	726
$[C_6F_5\text{-}I(Ph)]^+$	610
ZnF_2	442
$ZnCl_2$	357
$ZnBr_2$	442
ZnI_2	703
$Zn(C_6F_5)_2$	348
$[ZnCDipp(C_6F_5)]^+$	638
$[ZnCDippMe]^+$	582
$[ZnCDippEt]^+$	582
$TiCl_4$	384

2. GB–FIA model and correlation program

2.1. GB-FIA model and hypothesis test

To determine whether a new point (e.g., 400, 19.9) falls within the relevance curve established by GB-FIA model, a linear regression model can be used for fitting and hypothesis testing.

Step 1: Hypothesis

First, we need to clarify the hypothesis.

Null hypothesis (H_0): The new point (e.g., 400, 19.9) is on the relevance curve established by GB-FIA.

Alternative hypothesis (H_1): The new point (e.g., 400, 19.9) is not on the relevance curve established by GB-FIA.

Step 2: Regression Model

With the known data points FIA and GB, a linear regression model can be fitted to describe their relationship. This can be done using the least squares method.

Step 3: Calculate Residuals

After fitting the model, calculate the residuals for each data point on the fit curve (i.e., the difference between the actual observed values and the predicted values from the model). Residuals indicate the degree of deviation of each data point relative to the fit line.

Step 4: Hypothesis Test

Use the fitted model to calculate the predicted value of the new point (400, 19.9) and its residual. Then, perform hypothesis testing using the distribution of these residuals.

The key in hypothesis testing is calculating the confidence interval, which is a range containing the probable estimates of the true parameter. If the residual of the new point falls within this confidence interval, we cannot reject the null hypothesis.

Step 5: Determine

Compare the residual of the new point with the confidence interval. If the residual of the new point falls within the confidence interval, the null hypothesis cannot be rejected, implying that the new point could be on the relevance curve established by GB-FIA. If the residual falls outside the confidence interval, reject the null hypothesis, implying that the new point is not on the relevance curve established by GB-FIA.

2.2. Matlab program for establishing the GB-FIA model and hypothesis test

The Matlab program attached after this section is used to establish the GB-FIA regression model. The data used for modeling is from section 2.1, but there are some slight differences in the correlation between GB and FIA with these data. When the dataset is multi-compound dataset (large dataset), the correlation coefficient R2 between GB and FIA is 0.923. In contrast, when the dataset only includes Al compounds, the correlation coefficient R2 between GB and FIA is as high as 0.990. This indicates a strong correlation between the experimental GB of Al compounds, the calculated FIA, and the molecular model constructed for FIA calculations.

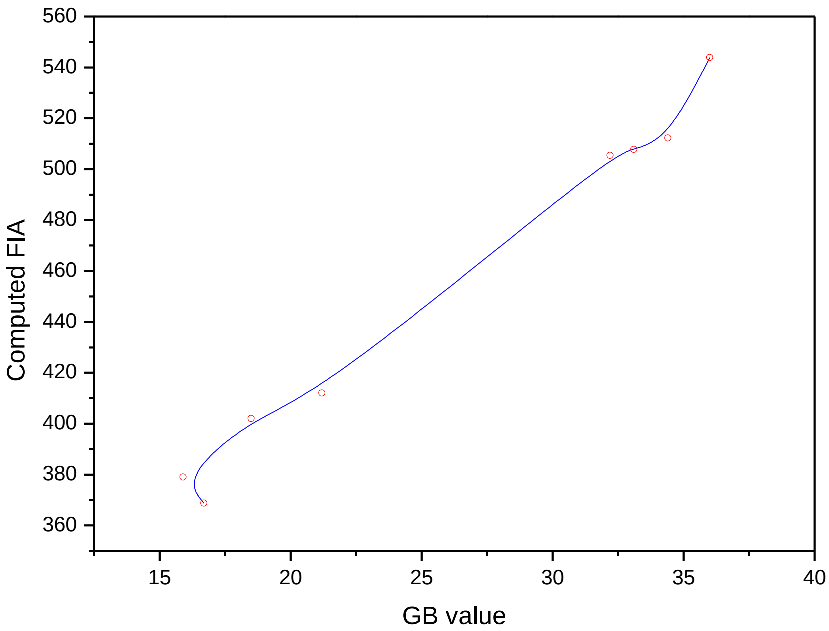

Figure S1. The correlation model established by Al-compound GB-FIA database.

Matlab program for establishing the GB-FIA model and hypothesis test:

```
function calfia
FIA=[368.8 379 402 412 505.4 507.8 512.2 543.8]';
GB=[16.7 15.9 18.5 21.2 32.2 33.1 34.4 36]';
n = length(GB);
p=polyfit(GB,FIA,2)
beta00=p(3)
```

```
beta11=p(2)
beta22=p(1)
X = [ones(n, 1), GB, GB.^2]
Y = FIA
beta = (X' * X) \ (X' * Y)    % Calculate the regression coefficients.
nbeta=inv(X'*X)*X'*Y
beta0 = beta(1)
beta1 = beta(2)
beta2 = beta(3)
predicted_FIA = beta0 + beta1 * GB + beta2 * GB.^2; % Calculate the predicted value
residuals = FIA - predicted_FIA;    % Calculate the residuals.
new_FIA = 400;
% new_GB = 19.9;
new_GB=[23.1 34.5 34.8 35.0 35.3 35.7 37.0];
predicted_FIA_new = beta0 + beta1 * new_GB + beta2 * new_GB.^2; % Calculate the predicted value of the new data point.
[M,N]=size(new_GB)
for i=1:N
    if predicted_FIA_new(i) >500
        predicted_FIA_new(i)=predicted_FIA_new(i)+70;
    end
end
predicted_FIA_new'
residual_new = new_FIA - predicted_FIA_new    % Calculate the residual of the new data point.
alpha = 0.05;    % Confidence level.
residual_std = std(residuals);    % Calculate the standard deviation of residuals.
z_critical = norminv(1 - alpha/2)    % Critical values for a normal distribution.
confidence_interval = [predicted_FIA_new - z_critical * residual_std, predicted_FIA_new + z_critical * residual_std]    % Calculate the confidence interval.
```

3.31. P NMR spectra and GC chromatogram

3.1.31. P NMR spectra for AN calculation

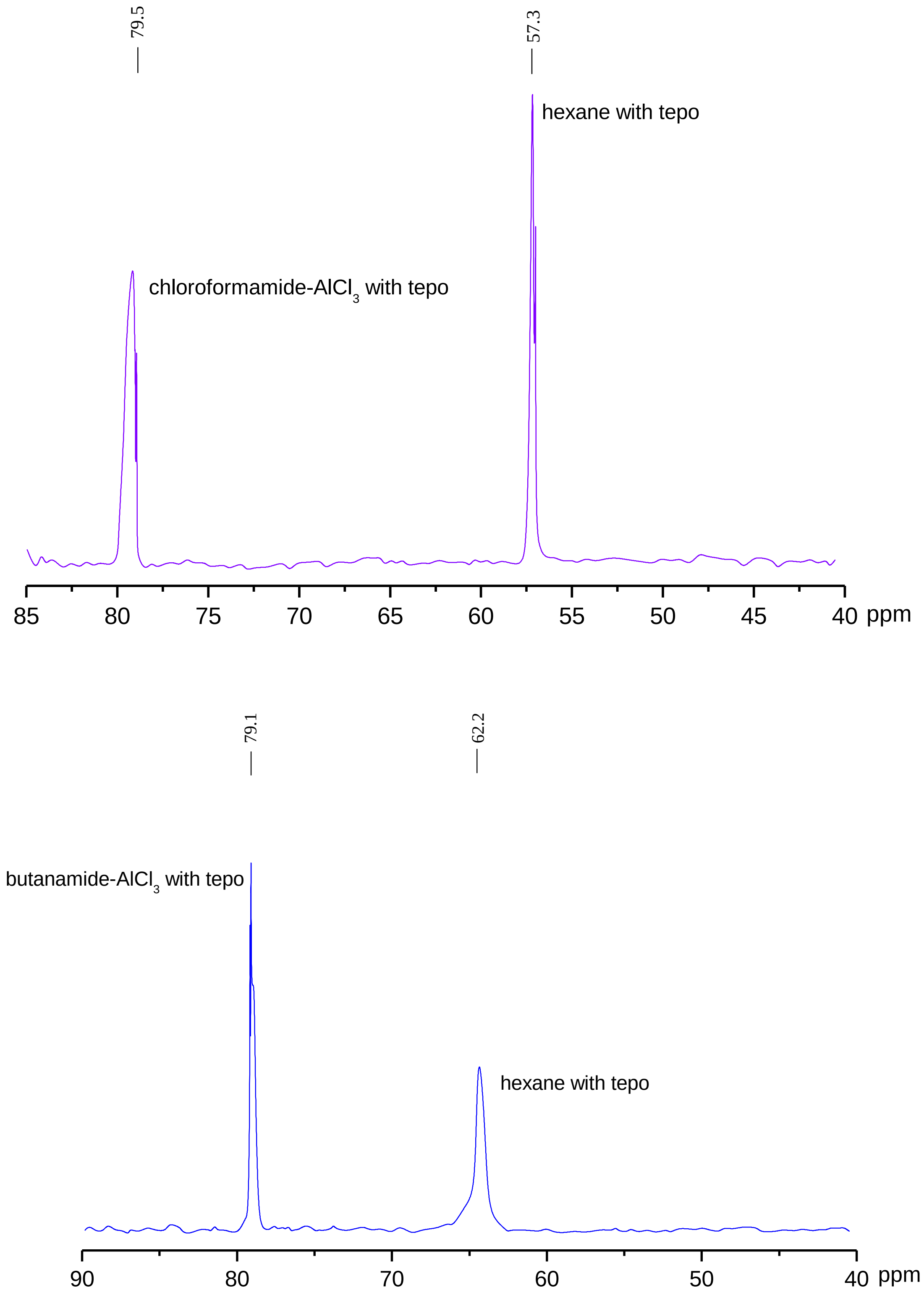

3.2.31. P NMR spectra for GB value calculation

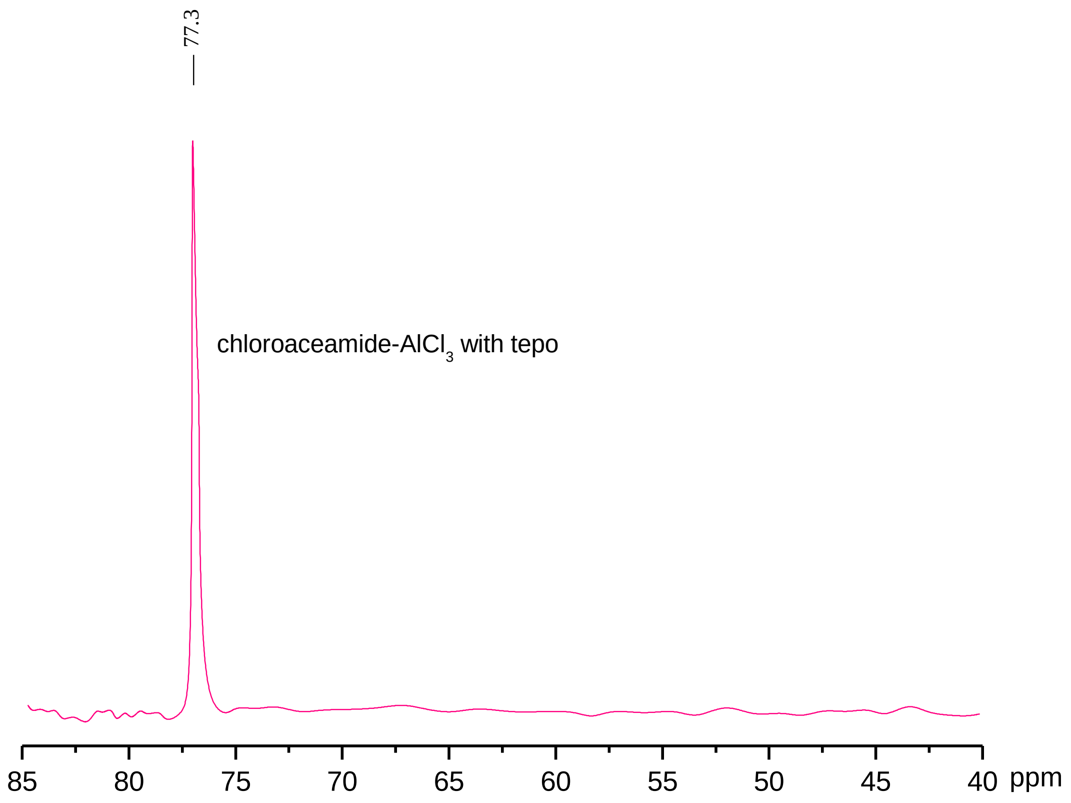
77.3
chloroaceamide-AlCl3 with tepo
85
80
75
70
65
60
55
50
45
40
ppm

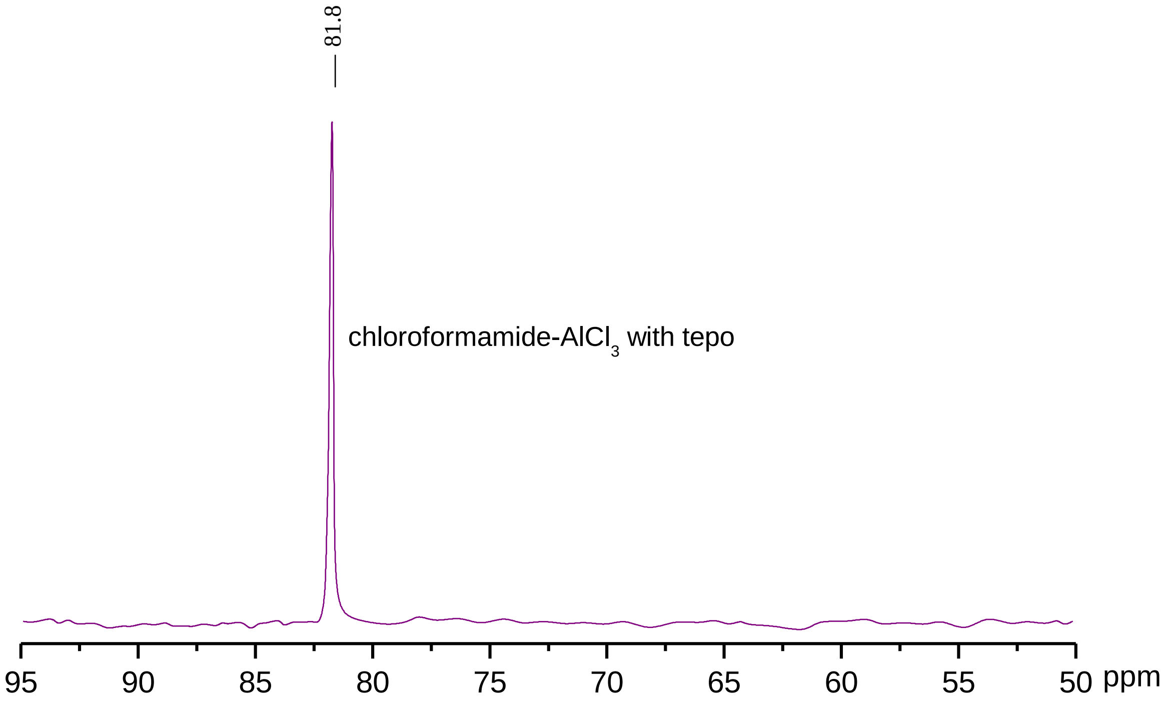
81.8
chloroformamide-AlCl3 with tepo
95
90
85
80
75
70
65
60
55
50
ppm

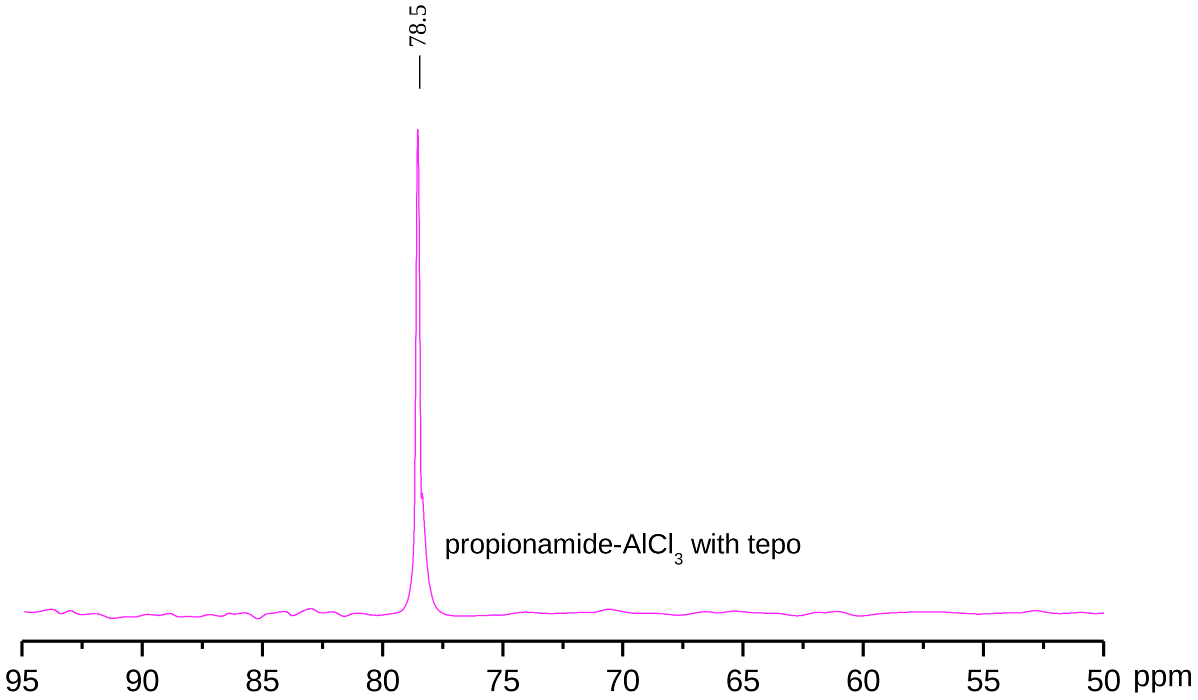

3.3. Typical GC chromatogram of the alkylate produced by acetamide-AlCl3 LC

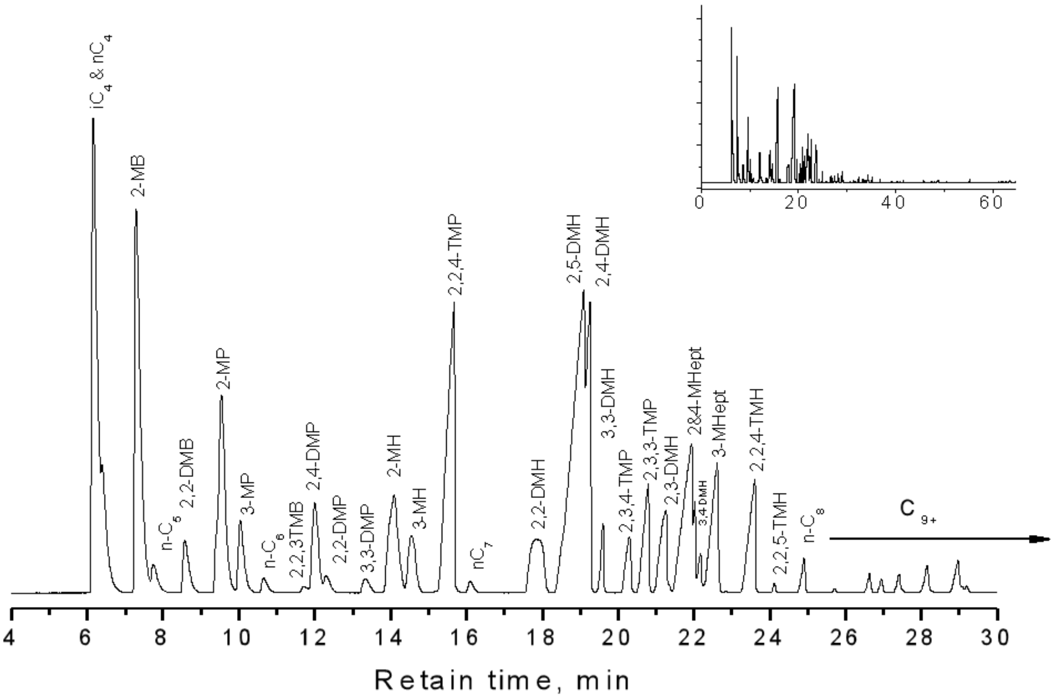

Supplement: Supplementary file 1 [file molecules-28-07857-s001.zip › molecules-2715449-supplementary.pdf]
